# Animacy or Case Marker Order?: Priority Information for Online Sentence Comprehension in a Head-Final Language

**DOI:** 10.1371/journal.pone.0093109

**Published:** 2014-03-24

**Authors:** Satoru Yokoyama, Kei Takahashi, Ryuta Kawashima

**Affiliations:** 1 Institute of Development, Aging, and Cancer, Tohoku University, Sendai, Miyagi, Japan; 2 Graduate School of International Cultural Studies, Tohoku University, Sendai, Miyagi, Japan; University of Leicester, United Kingdom

## Abstract

It is well known that case marker information and animacy information are incrementally used to comprehend sentences in head-final languages. However, it is still unclear how these two kinds of information are processed when they are in competition in a sentence's surface expression. The current study used sentences conveying the potentiality of some event (henceforth, potential sentences) in the Japanese language with theoretically canonical word order (dative–nominative/animate–inanimate order) and with scrambled word order (nominative–dative/inanimate–animate order). In Japanese, nominative–first case order and animate–inanimate animacy order are preferred to their reversed patterns in simplex sentences. Hence, in these potential sentences, case information and animacy information are in competition. The experiment consisted of a self-paced reading task testing two conditions (that is, canonical and scrambled potential sentences). Forty-five native speakers of Japanese participated. In our results, the canonical potential sentences showed a scrambling cost at the second argument position (the nominative argument). This result indicates that the theoretically scrambled case marker order (nominative–dative) is processed as a mentally canonical case marker order, suggesting that case information is used preferentially over animacy information when the two are in competition. The implications of our findings are discussed with regard to incremental simplex sentence comprehension models for head-final languages.

## Introduction

To comprehend a sentence, case processing is one of the essential linguistic processes that must be conducted [1]–[3]. In particular, for sentence comprehension in head-final languages, case marker processing is quite important to interpret the “who does what to whom” information or *thematic information* of a sentence [1], [3], [4]–[9]. For example, in Japanese, one such head-final language, an argument marked by nominative case marker *ga* tends to be interpreted as an actor [3], [4], whereas an argument marked by accusative case marker *o* tends to be interpreted as an undergoer [3], [4], [9]. Additionally, a dative-case-marked arguments will tend to be interpreted not only as an undergoer in transitive sentences, but also as an actor in dative-subject sentences [10].

The animacy information of arguments is also essential to interpret thematic information during sentence comprehension in head-final languages [3], [4], [9]. It is well known that semantically reversible sentences such as *the boy praised the girl* are more difficult to comprehend than the corresponding non-reversible sentences such as *the boy touched the table*, due to the animacy information provided by the arguments. It is widely assumed that this contrastive difference is caused by the greater number of possible interpretations of thematic role assignment of the subject and object in reversible sentences than of those in non-reversible sentences [9], [11]–[15].

So far, most experimental studies of these phenomena have investigated head-initial languages such as English in order to build a sentence comprehension theory or model. However, an increasing number of recent studies have come to focus on head-final languages. In terms of sentence processing strategies, researchers have claimed that some interesting differences exist between head-initial and head-final languages [3]. The most notable contrast is that whereas predicate/verb information can be used at an early stage of sentence comprehension in head-initial languages, it cannot be used until the end of sentence comprehension in head-final languages. This contrast leads us to speculate that whereas in head-initial languages, a predictive process of thematic interpretation of arguments is not required absolutely, such a predictive process is quite necessary in head-final languages. The reason is that in head-final languages, predicate information cannot be used until the input of the predicate at the end of the sentence, and hence if readers/listeners wait for that predicate input to interpret the sentence, a much larger short-term or working memory load is required to keep in memory all the argument information already input before the predicate. Thus, it can be assumed that arguments are incrementally processed before the predicate is input, to reduce this memory load. Much experimental research has provided evidence supporting this *incremental processing hypothesis* [3], [9], [16]–[21].

One previously proposed model of simplex sentence comprehension in head-final languages predicts that the case marker information and animacy information of arguments are incrementally used to process the thematic information of the sentence to which those arguments belong, before the predicate is input [3], [5]. Additionally, in the Japanese language, if a case marker exists in a sentence, case marker information will be used preferentially in this incremental processing. If no case marker exists (for example, in a situation of ellipsis or drop), then the animacy information of the arguments is used instead. In other words, case information has priority over animacy information as a tool for the thematic interpretation of arguments during simplex sentence comprehension [3], [4].

At this time, however, the empirical evidence for this priority is lacking. So far, there exists only one report regarding information priority in thematic processing in Japanese [4]. That study used an offline competition model paradigm and reported that the accusative argument tends to be thematically interpreted as an undergoer and the nominative argument as an actor. Additionally, when case markers do not appear explicitly, animate and inanimate nouns tend to be interpreted as undergoers and actors, respectively. Hence, this previous study concluded that case information has a higher priority for use in incremental thematic processing than animacy information does [4]. Recently, on the basis of results using an experimental online method, it was reported that animacy information is used even when a case marker appears on the surface expression of the sentence in Japanese. One recent experimental study reported that animacy information is incrementally used at the second argument position in transitive sentence comprehension, even when case markers appear on the sentence [9]. That study experimentally manipulated animacy information using reversibility a factor, but case marker information was not been manipulated. Hence, these findings can lead us to consider whether case marker information is used more preferentially than animacy information even when both types of information appear in the actual surface expression of the sentence and are in competition.

In the current study, we aim to examine whether case marker information or animacy information has priority during online sentence comprehension in Japanese. As stimuli, we used potential sentences, which consists of sentences conveying the potentiality of some event (henceforth, potential sentences). In potential sentences, case marker information and animacy information compete. In Japanese, the nominative argument is usually placed at the first argument position, and an animate noun is usually marked by a nominative case marker. Thus, an argument marked by dative case marker *ni*, for example, is interpreted as an undergoer, as in *John-ga Mary-ni kisushita* (John kissed Mary). In such a sentence, the nominative case marker usually marks an animate noun as an actor (e.g., *John-*NOM), which can be assumed to be preferred. In contrast, in a non-canonical use of the dative argument in a potential sentence, an argument marked by dative *ni* is interpreted as an actor, as in *John-ni Eigo-ga dekirudarouka* (Can John use English?). In this case, the nominative case marker marks an inanimate noun as an undergoer (*Eigo*-NOM). This situation has been pointed out in theoretical linguistic research [10].

Taking advantage of this competitive phenomenon in Japanese potential sentences, we prepared two types of experimental stimuli. The first one is potential sentences with dative-argument-first case marker order (henceforth, DAT-first order), and the second is potential sentences with nominative-argument-first case marker order (henceforth, NOM-first order). Examples are shown below (DAT: dative case marker, NOM: nominative case marker, POT: potential, AUX: auxiliary, Q: question). The character “/” denotes the sequence of the phrases in the actual stimuli presented in the current study.

1. Potential sentence with DAT-first order

sono-onnanoko-ni/sono-uta-ga/uta-eru-daroo-ka?

the girl-DAT the song-NOM sing-POT-AUX-Q.

‘Can the girl sing the song?’

(2) Potential sentence with NOM-first order

sono-uta-ga/sono-onnanoko-ni/uta-eru-daroo-ka?

the song-NOM the girl-DAT sing-POT-AUX-Q.

‘Can the girl sing the song?’

Our hypotheses according to previous findings are as follows. First of all, it has been consistently reported that scrambled sentences are more difficult to process than canonical ones in Japanese, and they show scrambling cost (e.g., longer reaction times) in behavior [9], [22]–[26]. Hence, sentences with canonical word order should be easier to process than the corresponding sentences with scrambled word order. If case marker order is used preferentially during Japanese sentence comprehension, sentences with NOM-first order should be canonical [9]. Compared with NOM-first order potential sentences, DAT-first order potential sentences would show a scrambling cost. Based on the recent Japanese simplex sentence comprehension model [3], in which it has been proposed that incremental thematic interpretation starts at the second argument position, the current experiment would show that the reading times of the second argument in the DAT-first potential sentences are longer than those in the NOM-first potential sentences because of a larger scrambling cost. In contrast, if animacy information is used preferentially, an argument with an animate noun as the first argument and argument with an inanimate noun as the second argument is preferred, since this order is a canonical order of sentences in Japanese [9]. In this case, DAT-first word order is a canonical word order. Hence, the current experiment would show that the reading times of the second argument in the NOM-first potential sentences are longer than those in the DAT-first potential sentences because of a larger scrambling cost. The patterns of case marker and animacy are summarized in Table 1.

**Table 1:** Patterns of case marking and animacy.

	First argument	Second argument
In Japanese		
Preferred case order	NOM	DAT
Preferred animacy order	Animate	Inanimate
In the current study		
NOM-first potential	Inanimate-NOM	Animate-DAT
DAT-first potential	Animate-DAT	Inanimate-NOM

## Methods

### Participants

Forty-five native Japanese speakers (thirteen female, mean age: 21.4, SD  = 1.3) participated in this experiment. Written informed consent was obtained from each subject, in accordance with the guidelines approved by the Medical School of Tohoku University on the basis of the Declaration of Helsinki, 2008. This experimental study was approved by the ethical committee of the Medical School of Tohoku University.

### Materials

Each target sentence consisted of two arguments (a noun marked by a nominative case particle and noun marked by a dative case particle), a verb with an auxiliary indicating potential, and an auxiliary verb such as *souda*, *youda*, and *rashii*, which convey the meaning of *seeming*. Additionally, all these potential sentences had an adverb representing temporal meaning such as *issyuukannde* (within a week). The animate nouns used were *sono-dansei*, *sono-josei*, *sono-otokonoko*, and *sono-onnanoko* (that man, that woman, that boy, and that girl, respectively) [9], [27]. The inanimate nouns were *uta* (song), *suugaku* (math), *bunsyou* (text), *biiru* (beer), *raamen* (ramen noodles), and similar high-frequency Japanese words. Using the above items, two types of stimulus sentence were created: NOM-first potential sentences and DAT-first potential sentences.

Eight target stimuli were prepared for each condition in the experiment. Additionally, 72 filler items were prepared in which sentences such as passive sentences and semantically reversible and non-reversible transitive sentences with accusative and dative arguments were included. All sentences did not have any ellipsis. The words used were high-frequency words similar to those used in the target sentences. The filler items were excluded from the data analysis.

### Procedure

The experiment was conducted using a notebook computer running Windows and the software E-prime 2.0 (PST Inc., Pittsburgh, PA). Participants were timed in a self-paced, non-cumulative, phrase-by-phrase moving-window reading task [28]. Stimuli initially appeared as dots with intervening spaces indicating the segments or regions of the sentence; participants pressed the “enter” key to reveal each subsequent region and cause the former region to revert to dots. At the end of each sentence, an additional sentence appeared on a new screen (e.g., for example (1) above, *sono-uta-ga sono-otokonoko-ni uta-eru-daroo-ka?* ‘Can the boy sing the song?’), and participants were asked to indicate whether the target stimulus and the additional sentence had the same meaning or not by pressing either “1” (yes) or “2” (no) on the keyboard. All sentences (i.e., target stimuli and yes/no question sentences) fit on a single line and were presented without line breaks. No feedback was provided. Before commencing the experiment, participants read written instructions and conducted eight practice trials. The experiment took participants approximately 30 minutes to complete on average.

## Results

With regard to the accuracy rates of the probe questions, an ANOVA showed no statistically significant difference between NOM-first and DAT-first potential sentences (*F*(1,44) = 0.75, *p* = 0.39).

Before performing the analysis of reading times, reaction times outside of 3.5 standard deviations (SD) at both the high and low ends of the range were normalized to the maximum or minimum boundary at 3.5 SD from the participant's individual mean in each condition, following the precedent of previous studies (Tamaoka et al. 2005; Yokoyama et al. 2013). This procedure affected less than 2% of all data.

The statistical tests analyzed both subject (F1) and item (F2) variability, which is a standard way to validate the statistical results [9], [18], [19], [25]. Only stimulus items for which participants provided correct responses were used in the analysis of reading times. To investigate our experimental hypotheses, we conducted the following statistical tests.

The first test was a 2×2 ANOVA of reading times between NOM-first and DAT-first potential sentences, with a sentence type factor (NOM-first vs. DAT-first) and an argument position factor (first vs. second argument position). We found no main effect of the sentence type factor (*F*1(1,44) = 3.5, *p* = 0.068, MSe(mean square error) = 135203.2; *F*2(1,15) = 3.4, *p* = 0.107, MSe = 19563.1). In contrast, we did find statistically longer reading times for the second argument than for the first argument (main effect of argument position, *F*1(1,44) = 29.8, *p*<0.001, MSe = 1178022.8; *F*2(1,15) = 107.4, *p*<0.001, MSe = 221088.6) as well as a statistically significant interaction between the sentence type and argument position (*F*1(1,44) = 6.9, *p*<0.05, MSe = 144969.2, *F*2(1,15) = 11.8, *p*<0.05, MSe = 28748.6). These results indicate that DAT-first potential sentences showed a greater scrambling cost at the second argument position than NOM-first potential sentences did.

The second test was a one-way ANOVA of reading times at the predicate position between NOM-first and DAT-first potential sentences, with the sentence type factor. At the predicate position, there is no position factor, because the number of predicate is one in the stimuli used in the current experiment. We found no main effect of this factor (*F*1(1,44) = 1.7, *p* = 0.19, MSe = 50276.0; *F*2(1,15) = 2.9, *p* = 0.12, MSe = 14757.2). This result indicates that there was no differential scrambling cost for potential sentence comprehension at the predicate position.

The accuracy rates and reading times described above are shown in Table 2.

**Table 2:** Behavioral data.

Accuracy rates	Mean	SD
Potential/DAT-first	89.1	14
Potential/NOM-first	87.7	12.3

Reading times	Mean	SD
1st argument		
Potential/DAT-first (DAT)	536	198
Potential/NOM-first (NOM)	537	177
2nd argument		
Potential/DAT-first (NOM)	754	360
Potential/NOM-first (DAT)	643	163
Predicate		
Potential/DAT-first	637	172
Potential/NOM-first	684	314

## Discussion

The purpose of the current study was to examine which takes priority during online sentence comprehension in Japanese, case marker information or animacy information, when these two are in competition. To this end, using a self-paced reading task, we compared reading times for argument and predicate positions between NOM-first and DAT-first potential sentences in Japanese. Previous studies have consistently reported that scrambled sentences are more difficult to process than canonical ones due to the presence of a scrambling cost [9], [22]–[26]. If case marker order is used preferentially during Japanese sentence comprehension, sentences with NOM-first order should be canonical [9]. Hence, in this case, compared with NOM-first order potential sentences, DAT-first order potential sentences would show a scrambling cost. In contrast, if animacy information is used preferentially, an argument with an animate noun as the first argument and argument with an inanimate noun as the second argument is preferred, since this order is a canonical order of sentences in Japanese [9]. In this case, DAT-first word order is a canonical word order. Hence, in this case, the reading times of the second argument in the NOM-first potential sentences would be longer than those in the DAT-first potential sentences because of a larger scrambling cost.

We conclude that case marker information is more preferentially used for argument processing in Japanese sentences than animacy information, on the basis of the experimental evidence that there was a statistically significant interaction between the sentence type factor and position factor (see Results). The result indicates that the second argument in DAT-first potential sentences shows a larger cost than in NOM-first potential sentences (see Table 2, Results). This means that greater scrambling-related cost (i.e., the interaction in our result) was observed in DAT-first potential sentences than NOM-first potential sentences. Hence, these results suggest that case marker order is used more preferentially to determine sentential canonicity than animacy information during Japanese sentence comprehension.

However, our results differ from the previous findings in terms of the relative canonicity of potential sentences [25]. Our results indicate that NOM-first potential sentences are processed as a canonical case marker order. In contrast, the previous study reported that DAT-first potential sentences are processed as a canonical order [25]. There is a threefold possible explanation for this discrepancy.

The first explanation relates to the use of a different experimental paradigm. In our experiment, we used a standard self-paced reading task, presented phrase-by-phrase, like the ones that have often been used in previous studies. In contrast, the previous study used a self-paced semantic decision task, with a whole sentence presented at once [25]. In this task, reaction times would include all processes related to processing, such as reading the arguments and the predicate, as well as the thinking time required to reach a semantic decision. Hence, it may be unclear exactly which process(es) the observed result reflects and to what degree. In contrast, with our task we can at least separate reading times for arguments and predicate. It will be recalled that we found different reading times at the second argument position. This suggests that task difference may cause the divergent results between the current study and the previous study [25].

The second possible explanation for these divergent results is that there is an effect of filler items; this is the more plausible explanation. In the current study, we used reversible and non-reversible transitive sentences with dative arguments as filler items (see Methods above). In contrast, the previous study seemingly did not adopt such filler items, since it was not noted in the paper [25]. In Japanese, dative transitive sentences are unmarked, whereas potential sentences are marked, in terms of case order. Hence, if such dative transitive sentences are not used as filler items, participants will be easily able to predict that the experiment will be presenting a potential sentence when they read a dative argument. The result observed in the previous study may be affected by this predictability [25].

The third possible explanation is that longer reading times of the second argument position in DAT-first potential sentences is caused by “a surprising effect”. According to corpus-based data, Miyamoto and Nakamura pointed out that Japanese native speakers tend to interpret sentences which have a non-nominative argument as the first argument as structures including a null-subject (ellipsis or drop) [29]. Since the previous study used not a comprehension data but a production data, it is unclear whether their claim can apply our comprehension data or not. If their claim is correct, verbs are input after the non-nominative argument. Hence, if a nominative argument is input after the non-nominative argument (dative argument in the current study), participants might be surprised, and it might cause longer reading times of the nominative argument following the non-nominative argument. This possible explanation conflicts with Tamaoka et al. 's results, and also with an explanation of results in reading potential sentences by a concept of scrambling cost or canonicity. However, at least, our conclusion is consistent with their claim. If Japanese native speakers interpret sentences which have a non-nominative argument as the first argument as structures including a null-subject, they should use case marker information more preferentially than animacy information. In potential sentences, animate noun is marked by a dative case marker. Hence, if Japanese native speakers use animacy information more preferentially than case marker information, Japanese native speakers should not interpret sentences which have a dative (animate) argument as the first argument as structures including a null-subject, but rather, interpret a dative argument as a first argument, because the dative argument is an animate noun. In this case, a nominative argument should not cause longer reading times in DAT-first potential sentences, since there is no surprising effect.

Finally, we would like to discuss a contribution that our findings make to sentence comprehension models targeting head-final languages. For these languages, particularly Japanese, case marker, animacy, and word order information are assumed to be information to incrementally interpret thematic information in sentences [3], [4]. These models assume that if case marker information is explicitly expressed in a sentence, it will be used to incrementally predict the thematic information of the sentence before input of the predicate, and that if case markers are not expressed in the sentence, the animacy information of the arguments will be used instead. If neither case marker nor animacy information is available, word order is used instead. However, to date, no one has clarified whether case markers and/or animacy are used for thematic processing in on-line sentence comprehension when both case marker and animacy are explicitly expressed in a sentence. To our knowledge, a recent experimental study has reported that animacy is used to incrementally interpret the thematic information of sentences even when case markers are explicitly expressed in the sentence but in no competition [9]. The current study newly provides evidence that case marker information is more preferentially used to process sentences than animacy information, even when case marker information and animacy information are both explicitly expressed and even in competition in the sentence.
